# Di-μ_2_-methano­lato-bis­(μ-4-methyl-5-sulfanyl­idene-4,5-dihydro-1*H*-1,2,4-triazolido-κ^2^ *N* ^1^:*N* ^2^)di-μ_3_-oxido-tetra­kis­[dibutyltin(IV)]

**DOI:** 10.1107/S1600536811001917

**Published:** 2011-01-22

**Authors:** Ezzatollah Najafi, Mostafa M. Amini, Seik Weng Ng

**Affiliations:** a Department of Chemistry, General Campus, Shahid Beheshti University, Tehran 1983963113, Iran; b Department of Chemistry, University of Malaya, 50603 Kuala Lumpur, Malaysia

## Abstract

The asymmetric unit of the title distannoxane, [Sn_4_(C_4_H_9_)_8_(C_3_H_4_N_3_S)_2_(CH_3_O)_2_O_2_], contains two mol­ecules, each of which lies about an individual center of inversion. The tetra­nuclear mol­ecule features a three-rung-staircase Sn_4_O_4_ core in which two independent Sn^IV^ atoms are bridged by the triazolide group. The negatively charged N atom of the triazolide group binds to the terminal Sn atom at a shorter distance  [Sn—N = 2.262 (3), 2.254 (3) Å] compared with the neutral N atom that binds to the central Sn atom[Sn
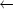
N = 2.617 (4); 2.830 (3) Å]. The oxide O atom is three-coordinate whereas the methano­late O atom is two-coordinate. The terminal Sn atom is five-coordinate in a *cis*-C_3_SnNO trigonal–bipyramidal environment, whereas the central Sn atom is six-coordinate in a C_2_SnNO_3_ skew-trapezoidal–bipyramidal geometry.

## Related literature

For background and a similar distannoxane, see: Najafi *et al.* (2011 ▶).
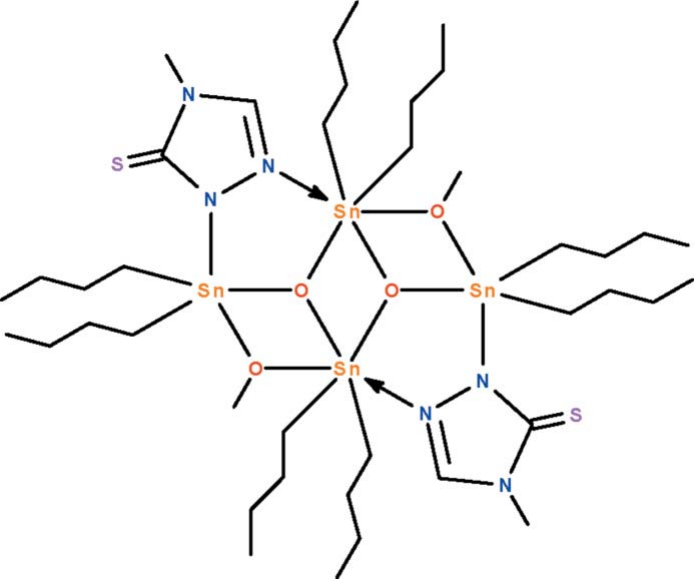


## Experimental

### 

#### Crystal data

[Sn_4_(C_4_H_9_)_8_(C_3_H_4_N_3_S)_2_(CH_3_O)_2_O_2_]
*M* *_r_* = 1254.03
Triclinic, 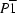

*a* = 12.3387 (3) Å
*b* = 12.9885 (4) Å
*c* = 16.7968 (5) Å
α = 81.437 (3)°
β = 83.636 (2)°
γ = 88.474 (2)°
*V* = 2645.33 (13) Å^3^
*Z* = 2
Mo *K*α radiation
μ = 1.99 mm^−1^
*T* = 100 K
0.30 × 0.30 × 0.10 mm

#### Data collection

Agilent SuperNova Dual diffractometer with an Atlas detector
Absorption correction: multi-scan (*CrysAlis PRO*; Agilent Technologies, 2010 ▶) *T*_min_ = 0.587, *T*_max_ = 0.826
21047 measured reflections
11731 independent reflections
9170 reflections with *I* > 2σ(*I*)
*R*_int_ = 0.038

#### Refinement

*R*[*F* ^2^ > 2σ(*F* ^2^)] = 0.037
*wR*(*F* ^2^) = 0.078
*S* = 1.01
11731 reflections
509 parameters
H-atom parameters constrained
Δρ_max_ = 0.74 e Å^−3^
Δρ_min_ = −0.99 e Å^−3^

### 

Data collection: *CrysAlis PRO* (Agilent Technologies, 2010 ▶); cell refinement: *CrysAlis PRO*; data reduction: *CrysAlis PRO*; program(s) used to solve structure: *SHELXS97* (Sheldrick, 2008 ▶); program(s) used to refine structure: *SHELXL97* (Sheldrick, 2008 ▶); molecular graphics: *X-SEED* (Barbour, 2001 ▶); software used to prepare material for publication: *publCIF* (Westrip, 2010 ▶).

## Supplementary Material

Crystal structure: contains datablocks global, I. DOI: 10.1107/S1600536811001917/xu5142sup1.cif

Structure factors: contains datablocks I. DOI: 10.1107/S1600536811001917/xu5142Isup2.hkl

Additional supplementary materials:  crystallographic information; 3D view; checkCIF report

## Figures and Tables

**Fig. 1.**
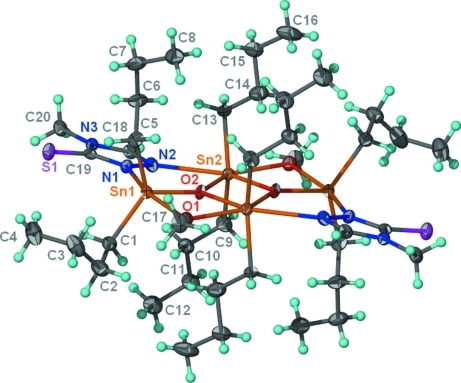
Thermal ellipsoid plot (Barbour, 2001) of one of the two molecules of Sn4O2(C4H9)8(CH3O)2(C3H4N3S)2 at the 70% probability level; hydrogen atoms are drawn as spheres of arbitrary radius.

**Fig. 2.**
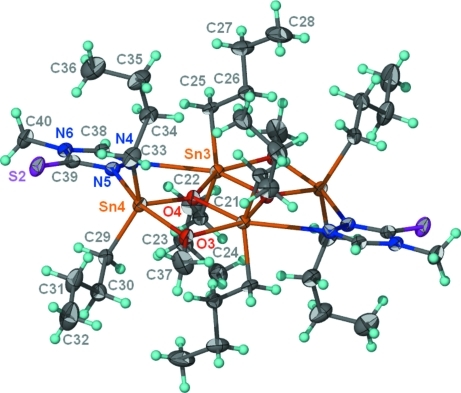
Thermal ellipsoid plot (Barbour, 2001) of the second molecule of Sn4O2(C4H9)8(CH3O)2(C3H4N3S)2 at the 70% probability level; hydrogen atoms are drawn as spheres of arbitrary radius.

## supplementary crystallographic information

### Comment

The preceding report on tetranuclear Sn_4_O_2_(CH_3_)_8_(CH_3_O)_2_(C_3_H_4_N_3_S)_2_ provides a short background to distannoxanes (Najafi *et al.*, 2011).

### Experimental

Dibutyltin diisothiocyanate (1 mmol), 4-methyl-4*H*-1,2,4-triazole-3-thiol (1 mmol) and 1,10-phenanthroline (1 mmol) were loaded into a convection tube; several drops of triethylamine were added. The tube was filled with dry methanol and kept at 333 K. Colorless crystals were collected from the side arm after several days.

### Refinement

Carbon-bound H-atoms were placed in calculated positions [C—H 0.95 to 0.98 Å, *U*_iso_(H) 1.2 to 1.5*U*_eq_(C)] and were included in the refinement in the riding model approximation.

### Crystal data

**Table:**

[Sn_4_(C_4_H_9_)_8_(C_3_H_4_N_3_S)_2_(CH_3_O)_2_O_2_]	*Z* = 2
*M**_r_* = 1254.03	*F*(000) = 1264
Triclinic, *P*1	*D*_x_ = 1.574 Mg m^−^^3^
Hall symbol: -P 1	Mo *K*α radiation, λ = 0.71073 Å
*a* = 12.3387 (3) Å	Cell parameters from 9821 reflections
*b* = 12.9885 (4) Å	θ = 2.3–29.3°
*c* = 16.7968 (5) Å	µ = 1.99 mm^−^^1^
α = 81.437 (3)°	*T* = 100 K
β = 83.636 (2)°	Block, colorless
γ = 88.474 (2)°	0.30 × 0.30 × 0.10 mm
*V* = 2645.33 (13) Å^3^	

### Data collection

**Table:**

Agilent SuperNova Dual diffractometer with an Atlas detector	11731 independent reflections
Radiation source: SuperNova (Mo) X-ray Source	9170 reflections with *I* > 2σ(*I*)
Mirror	*R*_int_ = 0.038
Detector resolution: 10.4041 pixels mm^-1^	θ_max_ = 27.5°, θ_min_ = 2.3°
ω scans	*h* = −12→15
Absorption correction: multi-scan (*CrysAlis PRO*; Agilent Technologies, 2010)	*k* = −15→16
*T*_min_ = 0.587, *T*_max_ = 0.826	*l* = −21→18
21047 measured reflections	

### Refinement

**Table:**

Refinement on *F*^2^	Primary atom site location: structure-invariant direct methods
Least-squares matrix: full	Secondary atom site location: difference Fourier map
*R*[*F*^2^ > 2σ(*F*^2^)] = 0.037	Hydrogen site location: inferred from neighbouring sites
*wR*(*F*^2^) = 0.078	H-atom parameters constrained
*S* = 1.01	*w* = 1/[σ^2^(*F*_o_^2^) + (0.0238*P*)^2^] where *P* = (*F*_o_^2^ + 2*F*_c_^2^)/3
11731 reflections	(Δ/σ)_max_ = 0.001
509 parameters	Δρ_max_ = 0.74 e Å^−^^3^
0 restraints	Δρ_min_ = −0.99 e Å^−^^3^

### Fractional atomic coordinates and isotropic or equivalent isotropic displacement parameters (Å^2^)

**Table:**

	*x*	*y*	*z*	*U*_iso_*/*U*_eq_	
Sn1	1.18487 (2)	0.65276 (2)	0.542466 (16)	0.01418 (7)	
Sn2	0.923401 (19)	0.58941 (2)	0.448691 (16)	0.01371 (7)	
Sn3	0.42542 (2)	1.04369 (2)	0.077415 (16)	0.01592 (7)	
Sn4	0.68464 (2)	0.86991 (2)	0.098980 (16)	0.01738 (7)	
S1	1.24656 (8)	0.93891 (9)	0.47383 (6)	0.0213 (2)	
S2	0.76555 (8)	0.83236 (10)	0.30111 (7)	0.0273 (3)	
O1	1.2076 (2)	0.4984 (2)	0.60264 (16)	0.0183 (6)	
O2	1.05994 (19)	0.5716 (2)	0.51529 (15)	0.0147 (6)	
O3	0.2937 (2)	1.1489 (2)	0.02857 (16)	0.0211 (7)	
O4	0.56314 (19)	0.9523 (2)	0.04735 (15)	0.0165 (6)	
N1	1.0989 (2)	0.7869 (3)	0.47233 (18)	0.0150 (7)	
N2	0.9958 (2)	0.7772 (3)	0.44781 (19)	0.0170 (7)	
N3	1.0462 (2)	0.9402 (3)	0.41771 (18)	0.0148 (7)	
N4	0.5004 (2)	0.9605 (3)	0.2273 (2)	0.0184 (8)	
N5	0.6059 (2)	0.9205 (3)	0.21464 (19)	0.0174 (7)	
N6	0.5618 (2)	0.9086 (3)	0.3441 (2)	0.0193 (8)	
C1	1.1579 (3)	0.7243 (3)	0.6502 (2)	0.0195 (9)	
H1A	1.1695	0.8001	0.6350	0.023*	
H1B	1.0807	0.7142	0.6726	0.023*	
C2	1.2298 (3)	0.6839 (4)	0.7169 (3)	0.0272 (10)	
H2A	1.2053	0.7166	0.7655	0.033*	
H2B	1.2198	0.6078	0.7316	0.033*	
C3	1.3501 (3)	0.7055 (4)	0.6931 (3)	0.0323 (12)	
H3A	1.3744	0.6726	0.6446	0.039*	
H3B	1.3913	0.6721	0.7376	0.039*	
C4	1.3788 (4)	0.8210 (4)	0.6750 (3)	0.0340 (12)	
H4A	1.4575	0.8282	0.6594	0.051*	
H4B	1.3581	0.8539	0.7234	0.051*	
H4C	1.3392	0.8548	0.6305	0.051*	
C5	1.3287 (3)	0.6515 (3)	0.4577 (2)	0.0200 (9)	
H5A	1.3856	0.6929	0.4752	0.024*	
H5B	1.3563	0.5790	0.4597	0.024*	
C6	1.3126 (3)	0.6940 (4)	0.3709 (2)	0.0246 (10)	
H6A	1.2975	0.7697	0.3668	0.029*	
H6B	1.2480	0.6608	0.3559	0.029*	
C7	1.4117 (3)	0.6757 (4)	0.3101 (3)	0.0282 (11)	
H7A	1.4036	0.7204	0.2579	0.034*	
H7B	1.4784	0.6975	0.3305	0.034*	
C8	1.4260 (3)	0.5649 (4)	0.2954 (3)	0.0287 (11)	
H8A	1.4907	0.5589	0.2568	0.043*	
H8B	1.3615	0.5434	0.2731	0.043*	
H8C	1.4351	0.5201	0.3465	0.043*	
C9	0.7907 (3)	0.6298 (4)	0.5314 (3)	0.0233 (10)	
H9A	0.7286	0.6487	0.4993	0.028*	
H9B	0.7700	0.5653	0.5687	0.028*	
C10	0.7979 (3)	0.7133 (4)	0.5830 (3)	0.0239 (10)	
H10A	0.8094	0.7809	0.5474	0.029*	
H10B	0.8623	0.6991	0.6134	0.029*	
C11	0.6985 (3)	0.7221 (4)	0.6421 (3)	0.0275 (11)	
H11A	0.6870	0.6543	0.6774	0.033*	
H11B	0.6343	0.7360	0.6114	0.033*	
C12	0.7036 (3)	0.8056 (4)	0.6948 (3)	0.0332 (12)	
H12A	0.6360	0.8057	0.7315	0.050*	
H12B	0.7123	0.8736	0.6607	0.050*	
H12C	0.7657	0.7919	0.7266	0.050*	
C13	1.0015 (3)	0.6182 (3)	0.3281 (2)	0.0167 (9)	
H13A	0.9519	0.6597	0.2931	0.020*	
H13B	1.0679	0.6597	0.3274	0.020*	
C14	1.0331 (3)	0.5177 (4)	0.2940 (3)	0.0279 (10)	
H14A	1.0724	0.4715	0.3338	0.033*	
H14B	0.9656	0.4818	0.2872	0.033*	
C15	1.1037 (3)	0.5336 (4)	0.2138 (3)	0.0267 (10)	
H15A	1.1717	0.5688	0.2203	0.032*	
H15B	1.0648	0.5796	0.1736	0.032*	
C16	1.1326 (4)	0.4311 (4)	0.1821 (3)	0.0396 (13)	
H16A	1.1790	0.4449	0.1305	0.059*	
H16B	1.0656	0.3971	0.1739	0.059*	
H16C	1.1717	0.3855	0.2215	0.059*	
C17	1.3064 (3)	0.4541 (3)	0.6300 (3)	0.0243 (10)	
H17A	1.3014	0.3780	0.6386	0.036*	
H17B	1.3179	0.4781	0.6810	0.036*	
H17C	1.3677	0.4757	0.5892	0.036*	
C18	0.9673 (3)	0.8695 (3)	0.4160 (2)	0.0186 (9)	
H18	0.9000	0.8858	0.3943	0.022*	
C19	1.1290 (3)	0.8865 (3)	0.4538 (2)	0.0155 (8)	
C20	1.0447 (3)	1.0492 (3)	0.3845 (2)	0.0210 (9)	
H20A	1.0779	1.0895	0.4202	0.031*	
H20B	0.9692	1.0726	0.3801	0.031*	
H20C	1.0860	1.0595	0.3307	0.031*	
C21	0.3130 (3)	0.9228 (3)	0.1234 (2)	0.0200 (9)	
H21A	0.2787	0.9035	0.0772	0.024*	
H21B	0.3548	0.8613	0.1459	0.024*	
C22	0.2224 (3)	0.9443 (4)	0.1879 (3)	0.0303 (11)	
H22A	0.1820	1.0080	0.1676	0.036*	
H22B	0.2550	0.9574	0.2367	0.036*	
C23	0.1424 (3)	0.8541 (4)	0.2114 (3)	0.0323 (11)	
H23A	0.1818	0.7922	0.2362	0.039*	
H23B	0.0848	0.8730	0.2528	0.039*	
C24	0.0888 (3)	0.8257 (4)	0.1400 (3)	0.0325 (12)	
H24A	0.0382	0.7682	0.1591	0.049*	
H24B	0.1451	0.8045	0.0996	0.049*	
H24C	0.0488	0.8863	0.1156	0.049*	
C25	0.5035 (3)	1.1760 (3)	0.1050 (2)	0.0195 (9)	
H25A	0.4523	1.2125	0.1413	0.023*	
H25B	0.5674	1.1527	0.1342	0.023*	
C26	0.5408 (3)	1.2510 (3)	0.0292 (2)	0.0211 (9)	
H26A	0.5900	1.2132	−0.0075	0.025*	
H26B	0.4764	1.2747	0.0010	0.025*	
C27	0.5997 (4)	1.3458 (4)	0.0450 (3)	0.0326 (12)	
H27A	0.6646	1.3229	0.0730	0.039*	
H27B	0.5508	1.3847	0.0811	0.039*	
C28	0.6351 (5)	1.4174 (4)	−0.0333 (3)	0.0491 (15)	
H28A	0.6736	1.4772	−0.0211	0.074*	
H28B	0.5708	1.4418	−0.0603	0.074*	
H28C	0.6839	1.3792	−0.0690	0.074*	
C29	0.6387 (3)	0.7131 (3)	0.1474 (3)	0.0221 (10)	
H29A	0.6478	0.7026	0.2059	0.027*	
H29B	0.5600	0.7066	0.1428	0.027*	
C30	0.6977 (3)	0.6249 (4)	0.1109 (3)	0.0260 (10)	
H30A	0.6861	0.6325	0.0529	0.031*	
H30B	0.6646	0.5584	0.1378	0.031*	
C31	0.8193 (4)	0.6188 (4)	0.1174 (3)	0.0373 (13)	
H31A	0.8528	0.6859	0.0923	0.045*	
H31B	0.8316	0.6076	0.1754	0.045*	
C32	0.8748 (4)	0.5320 (5)	0.0768 (3)	0.0525 (16)	
H32A	0.9529	0.5311	0.0827	0.079*	
H32B	0.8644	0.5436	0.0191	0.079*	
H32C	0.8430	0.4652	0.1021	0.079*	
C33	0.8267 (3)	0.9628 (4)	0.0912 (3)	0.0281 (11)	
H33A	0.8720	0.9331	0.1343	0.034*	
H33B	0.8702	0.9600	0.0384	0.034*	
C34	0.7995 (3)	1.0775 (4)	0.0999 (3)	0.0286 (11)	
H34A	0.7611	1.1092	0.0534	0.034*	
H34B	0.7493	1.0797	0.1498	0.034*	
C35	0.8997 (4)	1.1421 (4)	0.1035 (3)	0.0417 (14)	
H35A	0.8791	1.2168	0.0968	0.050*	
H35B	0.9553	1.1312	0.0582	0.050*	
C36	0.9487 (4)	1.1125 (5)	0.1842 (3)	0.0454 (14)	
H36A	1.0133	1.1549	0.1843	0.068*	
H36B	0.9697	1.0387	0.1908	0.068*	
H36C	0.8945	1.1251	0.2291	0.068*	
C37	0.2228 (4)	1.2117 (4)	0.0713 (3)	0.0402 (13)	
H37A	0.1531	1.2185	0.0481	0.060*	
H37B	0.2548	1.2807	0.0677	0.060*	
H37C	0.2107	1.1800	0.1282	0.060*	
C38	0.4776 (3)	0.9524 (3)	0.3048 (2)	0.0175 (9)	
H38	0.4106	0.9742	0.3310	0.021*	
C39	0.6432 (3)	0.8887 (3)	0.2857 (2)	0.0190 (9)	
C40	0.5666 (3)	0.8864 (4)	0.4313 (2)	0.0273 (11)	
H40A	0.5887	0.8137	0.4461	0.041*	
H40B	0.4945	0.8984	0.4595	0.041*	
H40C	0.6197	0.9323	0.4470	0.041*	

### Atomic displacement parameters (Å^2^)

**Table:**

	*U*^11^	*U*^22^	*U*^33^	*U*^12^	*U*^13^	*U*^23^
Sn1	0.01421 (14)	0.01295 (16)	0.01570 (15)	−0.00106 (10)	−0.00187 (10)	−0.00278 (12)
Sn2	0.01380 (13)	0.01229 (15)	0.01496 (15)	0.00039 (10)	−0.00094 (10)	−0.00233 (12)
Sn3	0.01557 (14)	0.01792 (17)	0.01427 (15)	0.00026 (11)	0.00060 (10)	−0.00407 (12)
Sn4	0.01548 (14)	0.02056 (17)	0.01681 (16)	0.00184 (11)	−0.00249 (10)	−0.00484 (13)
S1	0.0209 (5)	0.0181 (6)	0.0259 (6)	−0.0052 (4)	−0.0063 (4)	−0.0023 (5)
S2	0.0229 (6)	0.0362 (8)	0.0255 (6)	0.0081 (5)	−0.0103 (4)	−0.0089 (5)
O1	0.0204 (14)	0.0151 (16)	0.0193 (16)	0.0000 (12)	−0.0045 (11)	−0.0004 (13)
O2	0.0154 (13)	0.0092 (15)	0.0194 (15)	−0.0022 (11)	−0.0030 (11)	−0.0003 (12)
O3	0.0211 (14)	0.0259 (18)	0.0178 (16)	0.0060 (12)	−0.0056 (11)	−0.0070 (13)
O4	0.0155 (13)	0.0186 (17)	0.0154 (15)	0.0044 (11)	−0.0011 (10)	−0.0033 (12)
N1	0.0142 (16)	0.014 (2)	0.0162 (18)	−0.0019 (13)	−0.0031 (13)	−0.0006 (15)
N2	0.0147 (17)	0.019 (2)	0.0188 (19)	−0.0002 (14)	−0.0041 (13)	−0.0052 (16)
N3	0.0178 (17)	0.0121 (19)	0.0144 (18)	0.0004 (13)	−0.0026 (13)	−0.0013 (15)
N4	0.0177 (17)	0.017 (2)	0.020 (2)	0.0032 (14)	0.0003 (13)	−0.0044 (16)
N5	0.0179 (17)	0.019 (2)	0.0164 (19)	0.0034 (14)	0.0003 (13)	−0.0073 (15)
N6	0.0202 (18)	0.020 (2)	0.018 (2)	−0.0021 (15)	−0.0029 (14)	−0.0045 (16)
C1	0.021 (2)	0.021 (2)	0.018 (2)	−0.0003 (17)	−0.0032 (16)	−0.0038 (19)
C2	0.039 (3)	0.024 (3)	0.020 (2)	0.004 (2)	−0.0091 (19)	−0.004 (2)
C3	0.031 (3)	0.034 (3)	0.037 (3)	0.007 (2)	−0.015 (2)	−0.015 (2)
C4	0.030 (3)	0.033 (3)	0.045 (3)	0.002 (2)	−0.015 (2)	−0.014 (2)
C5	0.0114 (19)	0.024 (3)	0.024 (2)	−0.0010 (17)	−0.0012 (15)	−0.002 (2)
C6	0.030 (2)	0.022 (3)	0.018 (2)	0.0054 (19)	0.0037 (17)	0.002 (2)
C7	0.030 (2)	0.029 (3)	0.022 (3)	0.001 (2)	0.0085 (18)	0.000 (2)
C8	0.027 (2)	0.031 (3)	0.027 (3)	0.000 (2)	0.0041 (18)	−0.006 (2)
C9	0.014 (2)	0.031 (3)	0.027 (3)	0.0000 (18)	0.0031 (16)	−0.014 (2)
C10	0.024 (2)	0.027 (3)	0.022 (2)	−0.0005 (18)	0.0010 (17)	−0.009 (2)
C11	0.024 (2)	0.030 (3)	0.030 (3)	0.0035 (19)	0.0030 (18)	−0.012 (2)
C12	0.031 (3)	0.035 (3)	0.035 (3)	0.001 (2)	0.004 (2)	−0.013 (2)
C13	0.017 (2)	0.019 (2)	0.013 (2)	−0.0012 (16)	0.0015 (15)	−0.0020 (18)
C14	0.036 (3)	0.021 (3)	0.023 (3)	0.006 (2)	0.0043 (19)	0.000 (2)
C15	0.029 (2)	0.030 (3)	0.021 (2)	0.002 (2)	0.0003 (18)	−0.004 (2)
C16	0.058 (3)	0.038 (3)	0.020 (3)	0.018 (3)	0.006 (2)	−0.008 (2)
C17	0.025 (2)	0.020 (3)	0.028 (3)	0.0044 (18)	−0.0104 (18)	0.000 (2)
C18	0.020 (2)	0.019 (2)	0.017 (2)	−0.0004 (17)	−0.0040 (16)	−0.0048 (19)
C19	0.020 (2)	0.014 (2)	0.012 (2)	0.0012 (16)	0.0025 (15)	−0.0047 (17)
C20	0.030 (2)	0.012 (2)	0.021 (2)	0.0033 (17)	−0.0067 (17)	−0.0032 (19)
C21	0.024 (2)	0.019 (2)	0.017 (2)	−0.0032 (17)	0.0007 (16)	−0.0027 (19)
C22	0.026 (2)	0.034 (3)	0.030 (3)	−0.002 (2)	0.0050 (19)	−0.006 (2)
C23	0.031 (3)	0.030 (3)	0.032 (3)	−0.003 (2)	0.004 (2)	0.004 (2)
C24	0.028 (2)	0.028 (3)	0.039 (3)	−0.005 (2)	−0.005 (2)	0.004 (2)
C25	0.022 (2)	0.020 (2)	0.020 (2)	−0.0036 (17)	−0.0036 (16)	−0.0089 (19)
C26	0.022 (2)	0.021 (3)	0.020 (2)	−0.0012 (18)	−0.0017 (16)	−0.0041 (19)
C27	0.040 (3)	0.027 (3)	0.032 (3)	−0.010 (2)	0.002 (2)	−0.012 (2)
C28	0.076 (4)	0.027 (3)	0.043 (3)	−0.024 (3)	0.004 (3)	−0.005 (3)
C29	0.024 (2)	0.022 (3)	0.021 (2)	0.0020 (18)	−0.0060 (17)	−0.002 (2)
C30	0.029 (2)	0.026 (3)	0.023 (3)	0.0043 (19)	−0.0042 (18)	−0.004 (2)
C31	0.035 (3)	0.052 (4)	0.028 (3)	0.015 (2)	−0.013 (2)	−0.013 (3)
C32	0.056 (3)	0.066 (4)	0.040 (3)	0.036 (3)	−0.019 (3)	−0.020 (3)
C33	0.017 (2)	0.039 (3)	0.029 (3)	−0.0024 (19)	−0.0024 (17)	−0.007 (2)
C34	0.030 (2)	0.031 (3)	0.026 (3)	−0.003 (2)	−0.0035 (19)	−0.007 (2)
C35	0.041 (3)	0.049 (4)	0.040 (3)	−0.011 (3)	−0.011 (2)	−0.014 (3)
C36	0.038 (3)	0.055 (4)	0.047 (3)	−0.002 (3)	−0.007 (2)	−0.017 (3)
C37	0.048 (3)	0.039 (3)	0.030 (3)	0.014 (2)	0.008 (2)	−0.003 (3)
C38	0.015 (2)	0.019 (2)	0.019 (2)	−0.0005 (16)	−0.0014 (15)	−0.0047 (19)
C39	0.020 (2)	0.017 (2)	0.021 (2)	−0.0015 (17)	−0.0031 (16)	−0.0054 (19)
C40	0.030 (2)	0.035 (3)	0.018 (2)	0.001 (2)	−0.0061 (18)	−0.007 (2)

### Geometric parameters (Å, °)

**Table:**

Sn1—O2	2.020 (2)	C12—H12B	0.9800
Sn1—O1	2.133 (3)	C12—H12C	0.9800
Sn1—C1	2.144 (4)	C13—C14	1.527 (6)
Sn1—C5	2.150 (4)	C13—H13A	0.9900
Sn1—N1	2.262 (3)	C13—H13B	0.9900
Sn2—O2^i^	2.100 (3)	C14—C15	1.511 (6)
Sn2—O2	2.113 (2)	C14—H14A	0.9900
Sn2—C13	2.128 (4)	C14—H14B	0.9900
Sn2—C9	2.133 (4)	C15—C16	1.525 (6)
Sn2—O1^i^	2.315 (2)	C15—H15A	0.9900
Sn2—N2	2.617 (4)	C15—H15B	0.9900
Sn3—O4^ii^	2.078 (2)	C16—H16A	0.9800
Sn3—O4	2.106 (3)	C16—H16B	0.9800
Sn3—C21	2.122 (4)	C16—H16C	0.9800
Sn3—C25	2.126 (4)	C17—H17A	0.9800
Sn3—O3	2.242 (3)	C17—H17B	0.9800
Sn3—N4	2.830 (3)	C17—H17C	0.9800
Sn4—O4	2.018 (3)	C18—H18	0.9500
Sn4—C33	2.135 (4)	C20—H20A	0.9800
Sn4—C29	2.144 (4)	C20—H20B	0.9800
Sn4—O3^ii^	2.179 (3)	C20—H20C	0.9800
Sn4—N5	2.254 (3)	C21—C22	1.519 (6)
S1—C19	1.705 (4)	C21—H21A	0.9900
S2—C39	1.689 (4)	C21—H21B	0.9900
O1—C17	1.427 (4)	C22—C23	1.529 (6)
O1—Sn2^i^	2.315 (2)	C22—H22A	0.9900
O2—Sn2^i^	2.100 (3)	C22—H22B	0.9900
O3—C37	1.394 (5)	C23—C24	1.527 (6)
O3—Sn4^ii^	2.179 (3)	C23—H23A	0.9900
O4—Sn3^ii^	2.078 (2)	C23—H23B	0.9900
N1—C19	1.337 (5)	C24—H24A	0.9800
N1—N2	1.394 (4)	C24—H24B	0.9800
N2—C18	1.294 (5)	C24—H24C	0.9800
N3—C18	1.361 (5)	C25—C26	1.520 (5)
N3—C19	1.367 (5)	C25—H25A	0.9900
N3—C20	1.443 (5)	C25—H25B	0.9900
N4—C38	1.290 (5)	C26—C27	1.517 (5)
N4—N5	1.394 (4)	C26—H26A	0.9900
N5—C39	1.332 (5)	C26—H26B	0.9900
N6—C38	1.359 (5)	C27—C28	1.522 (6)
N6—C39	1.371 (5)	C27—H27A	0.9900
N6—C40	1.458 (5)	C27—H27B	0.9900
C1—C2	1.529 (5)	C28—H28A	0.9800
C1—H1A	0.9900	C28—H28B	0.9800
C1—H1B	0.9900	C28—H28C	0.9800
C2—C3	1.516 (6)	C29—C30	1.511 (6)
C2—H2A	0.9900	C29—H29A	0.9900
C2—H2B	0.9900	C29—H29B	0.9900
C3—C4	1.528 (6)	C30—C31	1.516 (6)
C3—H3A	0.9900	C30—H30A	0.9900
C3—H3B	0.9900	C30—H30B	0.9900
C4—H4A	0.9800	C31—C32	1.513 (7)
C4—H4B	0.9800	C31—H31A	0.9900
C4—H4C	0.9800	C31—H31B	0.9900
C5—C6	1.513 (5)	C32—H32A	0.9800
C5—H5A	0.9900	C32—H32B	0.9800
C5—H5B	0.9900	C32—H32C	0.9800
C6—C7	1.540 (6)	C33—C34	1.542 (6)
C6—H6A	0.9900	C33—H33A	0.9900
C6—H6B	0.9900	C33—H33B	0.9900
C7—C8	1.499 (6)	C34—C35	1.524 (5)
C7—H7A	0.9900	C34—H34A	0.9900
C7—H7B	0.9900	C34—H34B	0.9900
C8—H8A	0.9800	C35—C36	1.539 (6)
C8—H8B	0.9800	C35—H35A	0.9900
C8—H8C	0.9800	C35—H35B	0.9900
C9—C10	1.495 (6)	C36—H36A	0.9800
C9—H9A	0.9900	C36—H36B	0.9800
C9—H9B	0.9900	C36—H36C	0.9800
C10—C11	1.504 (5)	C37—H37A	0.9800
C10—H10A	0.9900	C37—H37B	0.9800
C10—H10B	0.9900	C37—H37C	0.9800
C11—C12	1.505 (6)	C38—H38	0.9500
C11—H11A	0.9900	C40—H40A	0.9800
C11—H11B	0.9900	C40—H40B	0.9800
C12—H12A	0.9800	C40—H40C	0.9800
			
O2—Sn1—O1	75.43 (10)	C15—C14—C13	114.2 (4)
O2—Sn1—C1	116.90 (13)	C15—C14—H14A	108.7
O1—Sn1—C1	96.07 (14)	C13—C14—H14A	108.7
O2—Sn1—C5	113.62 (13)	C15—C14—H14B	108.7
O1—Sn1—C5	95.34 (14)	C13—C14—H14B	108.7
C1—Sn1—C5	129.47 (14)	H14A—C14—H14B	107.6
O2—Sn1—N1	82.26 (10)	C14—C15—C16	112.2 (4)
O1—Sn1—N1	157.41 (10)	C14—C15—H15A	109.2
C1—Sn1—N1	90.76 (14)	C16—C15—H15A	109.2
C5—Sn1—N1	96.85 (14)	C14—C15—H15B	109.2
O2^i^—Sn2—O2	74.57 (10)	C16—C15—H15B	109.2
O2^i^—Sn2—C13	105.27 (13)	H15A—C15—H15B	107.9
O2—Sn2—C13	100.82 (12)	C15—C16—H16A	109.5
O2^i^—Sn2—C9	102.41 (14)	C15—C16—H16B	109.5
O2—Sn2—C9	105.39 (13)	H16A—C16—H16B	109.5
C13—Sn2—C9	145.94 (17)	C15—C16—H16C	109.5
O2^i^—Sn2—O1^i^	70.12 (9)	H16A—C16—H16C	109.5
O2—Sn2—O1^i^	144.63 (10)	H16B—C16—H16C	109.5
C13—Sn2—O1^i^	86.71 (12)	O1—C17—H17A	109.5
C9—Sn2—O1^i^	84.39 (12)	O1—C17—H17B	109.5
O4^ii^—Sn3—O4	74.11 (11)	H17A—C17—H17B	109.5
O4^ii^—Sn3—C21	104.11 (13)	O1—C17—H17C	109.5
O4—Sn3—C21	99.09 (14)	H17A—C17—H17C	109.5
O4^ii^—Sn3—C25	109.68 (13)	H17B—C17—H17C	109.5
O4—Sn3—C25	99.87 (13)	N2—C18—N3	111.3 (3)
C21—Sn3—C25	144.63 (15)	N2—C18—H18	124.4
O4^ii^—Sn3—O3	71.26 (10)	N3—C18—H18	124.4
O4—Sn3—O3	145.24 (10)	N1—C19—N3	107.3 (3)
C21—Sn3—O3	92.06 (14)	N1—C19—S1	126.8 (3)
C25—Sn3—O3	89.10 (13)	N3—C19—S1	125.8 (3)
O4^ii^—Sn3—N4	148.65 (10)	N3—C20—H20A	109.5
O4—Sn3—N4	74.78 (10)	N3—C20—H20B	109.5
C21—Sn3—N4	77.13 (12)	H20A—C20—H20B	109.5
C25—Sn3—N4	79.44 (13)	N3—C20—H20C	109.5
O3—Sn3—N4	139.97 (10)	H20A—C20—H20C	109.5
O4—Sn4—C33	111.02 (15)	H20B—C20—H20C	109.5
O4—Sn4—C29	112.53 (13)	C22—C21—Sn3	117.8 (3)
C33—Sn4—C29	136.45 (17)	C22—C21—H21A	107.9
O4—Sn4—O3^ii^	73.69 (10)	Sn3—C21—H21A	107.9
C33—Sn4—O3^ii^	94.32 (14)	C22—C21—H21B	107.9
C29—Sn4—O3^ii^	97.74 (14)	Sn3—C21—H21B	107.9
O4—Sn4—N5	84.74 (11)	H21A—C21—H21B	107.2
C33—Sn4—N5	95.20 (14)	C21—C22—C23	112.6 (4)
C29—Sn4—N5	88.63 (14)	C21—C22—H22A	109.1
O3^ii^—Sn4—N5	158.35 (11)	C23—C22—H22A	109.1
C17—O1—Sn1	126.7 (2)	C21—C22—H22B	109.1
C17—O1—Sn2^i^	125.6 (2)	C23—C22—H22B	109.1
Sn1—O1—Sn2^i^	100.70 (10)	H22A—C22—H22B	107.8
Sn1—O2—Sn2^i^	112.52 (11)	C24—C23—C22	113.5 (4)
Sn1—O2—Sn2	141.46 (14)	C24—C23—H23A	108.9
Sn2^i^—O2—Sn2	105.43 (10)	C22—C23—H23A	108.9
C37—O3—Sn4^ii^	131.6 (3)	C24—C23—H23B	108.9
C37—O3—Sn3	126.9 (3)	C22—C23—H23B	108.9
Sn4^ii^—O3—Sn3	101.40 (11)	H23A—C23—H23B	107.7
Sn4—O4—Sn3^ii^	113.30 (12)	C23—C24—H24A	109.5
Sn4—O4—Sn3	140.76 (13)	C23—C24—H24B	109.5
Sn3^ii^—O4—Sn3	105.89 (11)	H24A—C24—H24B	109.5
C19—N1—N2	108.7 (3)	C23—C24—H24C	109.5
C19—N1—Sn1	128.6 (2)	H24A—C24—H24C	109.5
N2—N1—Sn1	122.4 (2)	H24B—C24—H24C	109.5
C18—N2—N1	106.2 (3)	C26—C25—Sn3	111.9 (3)
C18—N3—C19	106.5 (3)	C26—C25—H25A	109.2
C18—N3—C20	126.6 (3)	Sn3—C25—H25A	109.2
C19—N3—C20	126.9 (3)	C26—C25—H25B	109.2
C38—N4—N5	105.8 (3)	Sn3—C25—H25B	109.2
C38—N4—Sn3	143.7 (3)	H25A—C25—H25B	107.9
N5—N4—Sn3	110.2 (2)	C27—C26—C25	114.2 (3)
C39—N5—N4	109.6 (3)	C27—C26—H26A	108.7
C39—N5—Sn4	121.9 (3)	C25—C26—H26A	108.7
N4—N5—Sn4	126.1 (2)	C27—C26—H26B	108.7
C38—N6—C39	106.8 (3)	C25—C26—H26B	108.7
C38—N6—C40	127.4 (4)	H26A—C26—H26B	107.6
C39—N6—C40	125.8 (3)	C26—C27—C28	111.5 (4)
C2—C1—Sn1	115.7 (3)	C26—C27—H27A	109.3
C2—C1—H1A	108.4	C28—C27—H27A	109.3
Sn1—C1—H1A	108.4	C26—C27—H27B	109.3
C2—C1—H1B	108.4	C28—C27—H27B	109.3
Sn1—C1—H1B	108.4	H27A—C27—H27B	108.0
H1A—C1—H1B	107.4	C27—C28—H28A	109.5
C3—C2—C1	113.7 (4)	C27—C28—H28B	109.5
C3—C2—H2A	108.8	H28A—C28—H28B	109.5
C1—C2—H2A	108.8	C27—C28—H28C	109.5
C3—C2—H2B	108.8	H28A—C28—H28C	109.5
C1—C2—H2B	108.8	H28B—C28—H28C	109.5
H2A—C2—H2B	107.7	C30—C29—Sn4	118.6 (3)
C2—C3—C4	114.5 (4)	C30—C29—H29A	107.7
C2—C3—H3A	108.6	Sn4—C29—H29A	107.7
C4—C3—H3A	108.6	C30—C29—H29B	107.7
C2—C3—H3B	108.6	Sn4—C29—H29B	107.7
C4—C3—H3B	108.6	H29A—C29—H29B	107.1
H3A—C3—H3B	107.6	C29—C30—C31	114.9 (4)
C3—C4—H4A	109.5	C29—C30—H30A	108.5
C3—C4—H4B	109.5	C31—C30—H30A	108.5
H4A—C4—H4B	109.5	C29—C30—H30B	108.5
C3—C4—H4C	109.5	C31—C30—H30B	108.5
H4A—C4—H4C	109.5	H30A—C30—H30B	107.5
H4B—C4—H4C	109.5	C32—C31—C30	112.5 (4)
C6—C5—Sn1	114.9 (3)	C32—C31—H31A	109.1
C6—C5—H5A	108.5	C30—C31—H31A	109.1
Sn1—C5—H5A	108.5	C32—C31—H31B	109.1
C6—C5—H5B	108.5	C30—C31—H31B	109.1
Sn1—C5—H5B	108.5	H31A—C31—H31B	107.8
H5A—C5—H5B	107.5	C31—C32—H32A	109.5
C5—C6—C7	113.4 (4)	C31—C32—H32B	109.5
C5—C6—H6A	108.9	H32A—C32—H32B	109.5
C7—C6—H6A	108.9	C31—C32—H32C	109.5
C5—C6—H6B	108.9	H32A—C32—H32C	109.5
C7—C6—H6B	108.9	H32B—C32—H32C	109.5
H6A—C6—H6B	107.7	C34—C33—Sn4	112.9 (3)
C8—C7—C6	113.9 (4)	C34—C33—H33A	109.0
C8—C7—H7A	108.8	Sn4—C33—H33A	109.0
C6—C7—H7A	108.8	C34—C33—H33B	109.0
C8—C7—H7B	108.8	Sn4—C33—H33B	109.0
C6—C7—H7B	108.8	H33A—C33—H33B	107.8
H7A—C7—H7B	107.7	C35—C34—C33	113.5 (4)
C7—C8—H8A	109.5	C35—C34—H34A	108.9
C7—C8—H8B	109.5	C33—C34—H34A	108.9
H8A—C8—H8B	109.5	C35—C34—H34B	108.9
C7—C8—H8C	109.5	C33—C34—H34B	108.9
H8A—C8—H8C	109.5	H34A—C34—H34B	107.7
H8B—C8—H8C	109.5	C34—C35—C36	111.6 (4)
C10—C9—Sn2	122.8 (3)	C34—C35—H35A	109.3
C10—C9—H9A	106.6	C36—C35—H35A	109.3
Sn2—C9—H9A	106.6	C34—C35—H35B	109.3
C10—C9—H9B	106.6	C36—C35—H35B	109.3
Sn2—C9—H9B	106.6	H35A—C35—H35B	108.0
H9A—C9—H9B	106.6	C35—C36—H36A	109.5
C9—C10—C11	113.8 (3)	C35—C36—H36B	109.5
C9—C10—H10A	108.8	H36A—C36—H36B	109.5
C11—C10—H10A	108.8	C35—C36—H36C	109.5
C9—C10—H10B	108.8	H36A—C36—H36C	109.5
C11—C10—H10B	108.8	H36B—C36—H36C	109.5
H10A—C10—H10B	107.7	O3—C37—H37A	109.5
C10—C11—C12	114.9 (4)	O3—C37—H37B	109.5
C10—C11—H11A	108.5	H37A—C37—H37B	109.5
C12—C11—H11A	108.5	O3—C37—H37C	109.5
C10—C11—H11B	108.5	H37A—C37—H37C	109.5
C12—C11—H11B	108.5	H37B—C37—H37C	109.5
H11A—C11—H11B	107.5	N4—C38—N6	111.3 (4)
C11—C12—H12A	109.5	N4—C38—H38	124.3
C11—C12—H12B	109.5	N6—C38—H38	124.3
H12A—C12—H12B	109.5	N5—C39—N6	106.5 (3)
C11—C12—H12C	109.5	N5—C39—S2	126.8 (3)
H12A—C12—H12C	109.5	N6—C39—S2	126.7 (3)
H12B—C12—H12C	109.5	N6—C40—H40A	109.5
C14—C13—Sn2	112.3 (3)	N6—C40—H40B	109.5
C14—C13—H13A	109.1	H40A—C40—H40B	109.5
Sn2—C13—H13A	109.1	N6—C40—H40C	109.5
C14—C13—H13B	109.1	H40A—C40—H40C	109.5
Sn2—C13—H13B	109.1	H40B—C40—H40C	109.5
H13A—C13—H13B	107.9		
			
O2—Sn1—O1—C17	159.7 (3)	O3^ii^—Sn4—N5—C39	172.0 (3)
C1—Sn1—O1—C17	−84.0 (3)	O4—Sn4—N5—N4	16.6 (3)
C5—Sn1—O1—C17	46.7 (3)	C33—Sn4—N5—N4	127.3 (3)
N1—Sn1—O1—C17	169.1 (3)	C29—Sn4—N5—N4	−96.1 (3)
O2—Sn1—O1—Sn2^i^	8.03 (10)	O3^ii^—Sn4—N5—N4	11.6 (5)
C1—Sn1—O1—Sn2^i^	124.30 (13)	O2—Sn1—C1—C2	116.3 (3)
C5—Sn1—O1—Sn2^i^	−105.01 (13)	O1—Sn1—C1—C2	39.6 (3)
N1—Sn1—O1—Sn2^i^	17.4 (3)	C5—Sn1—C1—C2	−62.4 (4)
O1—Sn1—O2—Sn2^i^	−9.43 (11)	N1—Sn1—C1—C2	−162.0 (3)
C1—Sn1—O2—Sn2^i^	−98.89 (17)	Sn1—C1—C2—C3	64.5 (4)
C5—Sn1—O2—Sn2^i^	80.07 (17)	C1—C2—C3—C4	63.0 (5)
N1—Sn1—O2—Sn2^i^	174.20 (14)	O2—Sn1—C5—C6	58.1 (3)
O1—Sn1—O2—Sn2	−178.8 (2)	O1—Sn1—C5—C6	134.6 (3)
C1—Sn1—O2—Sn2	91.8 (2)	C1—Sn1—C5—C6	−123.1 (3)
C5—Sn1—O2—Sn2	−89.3 (2)	N1—Sn1—C5—C6	−26.4 (3)
N1—Sn1—O2—Sn2	4.9 (2)	Sn1—C5—C6—C7	−170.6 (3)
O2^i^—Sn2—O2—Sn1	169.8 (3)	C5—C6—C7—C8	73.1 (5)
C13—Sn2—O2—Sn1	66.7 (2)	O2^i^—Sn2—C9—C10	125.7 (4)
C9—Sn2—O2—Sn1	−91.3 (2)	O2—Sn2—C9—C10	48.5 (4)
O1^i^—Sn2—O2—Sn1	166.42 (16)	C13—Sn2—C9—C10	−90.5 (4)
O2^i^—Sn2—O2—Sn2^i^	0.0	O1^i^—Sn2—C9—C10	−166.1 (4)
C13—Sn2—O2—Sn2^i^	−103.03 (14)	Sn2—C9—C10—C11	−174.8 (3)
C9—Sn2—O2—Sn2^i^	98.93 (15)	C9—C10—C11—C12	180.0 (4)
O1^i^—Sn2—O2—Sn2^i^	−3.3 (2)	O2^i^—Sn2—C13—C14	8.8 (3)
O4^ii^—Sn3—O3—C37	−173.1 (4)	O2—Sn2—C13—C14	85.5 (3)
O4—Sn3—O3—C37	−168.1 (3)	C9—Sn2—C13—C14	−134.5 (3)
C21—Sn3—O3—C37	82.7 (4)	O1^i^—Sn2—C13—C14	−59.6 (3)
C25—Sn3—O3—C37	−61.9 (4)	Sn2—C13—C14—C15	−171.1 (3)
N4—Sn3—O3—C37	10.4 (4)	C13—C14—C15—C16	−179.8 (4)
O4^ii^—Sn3—O3—Sn4^ii^	4.21 (10)	N1—N2—C18—N3	0.3 (4)
O4—Sn3—O3—Sn4^ii^	9.2 (2)	C19—N3—C18—N2	−0.3 (4)
C21—Sn3—O3—Sn4^ii^	−100.00 (14)	C20—N3—C18—N2	177.0 (4)
C25—Sn3—O3—Sn4^ii^	115.37 (14)	N2—N1—C19—N3	0.0 (4)
N4—Sn3—O3—Sn4^ii^	−172.32 (10)	Sn1—N1—C19—N3	−174.3 (2)
C33—Sn4—O4—Sn3^ii^	83.45 (17)	N2—N1—C19—S1	178.1 (3)
C29—Sn4—O4—Sn3^ii^	−96.67 (17)	Sn1—N1—C19—S1	3.9 (5)
O3^ii^—Sn4—O4—Sn3^ii^	−4.92 (11)	C18—N3—C19—N1	0.2 (4)
N5—Sn4—O4—Sn3^ii^	177.01 (14)	C20—N3—C19—N1	−177.2 (3)
C33—Sn4—O4—Sn3	−93.7 (2)	C18—N3—C19—S1	−177.9 (3)
C29—Sn4—O4—Sn3	86.2 (2)	C20—N3—C19—S1	4.7 (6)
O3^ii^—Sn4—O4—Sn3	177.9 (2)	O4^ii^—Sn3—C21—C22	−133.3 (3)
N5—Sn4—O4—Sn3	−0.1 (2)	O4—Sn3—C21—C22	150.9 (3)
O4^ii^—Sn3—O4—Sn4	177.3 (3)	C25—Sn3—C21—C22	29.2 (5)
C21—Sn3—O4—Sn4	−80.5 (2)	O3—Sn3—C21—C22	−62.2 (3)
C25—Sn3—O4—Sn4	69.5 (2)	N4—Sn3—C21—C22	78.9 (3)
O3—Sn3—O4—Sn4	172.33 (16)	Sn3—C21—C22—C23	175.9 (3)
N4—Sn3—O4—Sn4	−6.64 (19)	C21—C22—C23—C24	−57.6 (5)
O4^ii^—Sn3—O4—Sn3^ii^	0.0	O4^ii^—Sn3—C25—C26	5.8 (3)
C21—Sn3—O4—Sn3^ii^	102.18 (14)	O4—Sn3—C25—C26	82.4 (3)
C25—Sn3—O4—Sn3^ii^	−107.81 (14)	C21—Sn3—C25—C26	−156.1 (3)
O3—Sn3—O4—Sn3^ii^	−4.9 (2)	O3—Sn3—C25—C26	−63.9 (3)
N4—Sn3—O4—Sn3^ii^	176.09 (13)	N4—Sn3—C25—C26	154.7 (3)
O2—Sn1—N1—C19	−177.4 (3)	Sn3—C25—C26—C27	−178.6 (3)
O1—Sn1—N1—C19	173.4 (3)	C25—C26—C27—C28	179.8 (4)
C1—Sn1—N1—C19	65.5 (3)	O4—Sn4—C29—C30	115.8 (3)
C5—Sn1—N1—C19	−64.4 (3)	C33—Sn4—C29—C30	−64.4 (4)
O2—Sn1—N1—N2	9.1 (3)	O3^ii^—Sn4—C29—C30	40.3 (3)
O1—Sn1—N1—N2	−0.1 (5)	N5—Sn4—C29—C30	−160.5 (3)
C1—Sn1—N1—N2	−108.0 (3)	Sn4—C29—C30—C31	61.2 (5)
C5—Sn1—N1—N2	122.1 (3)	C29—C30—C31—C32	−177.6 (4)
C19—N1—N2—C18	−0.2 (4)	O4—Sn4—C33—C34	35.7 (3)
Sn1—N1—N2—C18	174.5 (3)	C29—Sn4—C33—C34	−144.1 (3)
O4^ii^—Sn3—N4—C38	−165.9 (4)	O3^ii^—Sn4—C33—C34	109.9 (3)
O4—Sn3—N4—C38	−173.2 (5)	N5—Sn4—C33—C34	−50.6 (3)
C21—Sn3—N4—C38	−69.9 (4)	Sn4—C33—C34—C35	173.9 (3)
C25—Sn3—N4—C38	83.5 (4)	C33—C34—C35—C36	−71.3 (5)
O3—Sn3—N4—C38	7.7 (5)	N5—N4—C38—N6	−0.5 (4)
O4^ii^—Sn3—N4—N5	22.3 (3)	Sn3—N4—C38—N6	−172.5 (3)
O4—Sn3—N4—N5	15.0 (2)	C39—N6—C38—N4	0.4 (5)
C21—Sn3—N4—N5	118.3 (3)	C40—N6—C38—N4	−179.8 (4)
C25—Sn3—N4—N5	−88.4 (2)	N4—N5—C39—N6	−0.2 (4)
O3—Sn3—N4—N5	−164.1 (2)	Sn4—N5—C39—N6	−163.5 (2)
C38—N4—N5—C39	0.4 (4)	N4—N5—C39—S2	178.5 (3)
Sn3—N4—N5—C39	175.4 (2)	Sn4—N5—C39—S2	15.2 (5)
C38—N4—N5—Sn4	162.8 (3)	C38—N6—C39—N5	−0.1 (4)
Sn3—N4—N5—Sn4	−22.2 (3)	C40—N6—C39—N5	−179.9 (4)
O4—Sn4—N5—C39	177.0 (3)	C38—N6—C39—S2	−178.8 (3)
C33—Sn4—N5—C39	−72.3 (3)	C40—N6—C39—S2	1.3 (6)
C29—Sn4—N5—C39	64.3 (3)		

Symmetry codes: (i) −*x*+2, −*y*+1, −*z*+1; (ii) −*x*+1, −*y*+2, −*z*.
